# Transcriptome Analysis of Chilling-Imbibed Embryo Revealed Membrane Recovery Related Genes in Maize

**DOI:** 10.3389/fpls.2016.01978

**Published:** 2017-01-04

**Authors:** Fei He, Hangqi Shen, Cheng Lin, Hong Fu, Mohamed S. Sheteiwy, Yajing Guan, Yutao Huang, Jin Hu

**Affiliations:** ^1^Seed Science Center, Institute of Crop Science, College of Agriculture and Biotechnology, Zhejiang UniversityHangzhou, China; ^2^Department of Agronomy, Faculty of Agriculture, Mansoura UniversityMansoura, Egypt

**Keywords:** chilling injury, imbibition, maize embryo, plasma membrane, transcriptome

## Abstract

The delayed seed germination and poor seedling growth caused by imbibitional chilling injury was common phenomenon in maize seedling establishment. In this study, RNA sequencing technology was used to comprehensively investigate the gene expressions in chilling-imbibed maize embryo and to reveal the underlying mechanism of chilling injury at molecular level. Imbibed seeds for 2 h at 5°C (LT2) were selected and transcriptomic comparative analysis was performed. Among 327 DEGs indentified between dry seed (CK0) and LT2, 15 specific genes with plasma membrane (PM) relevant functions belonging to lipid metabolism, stress, signaling and transport were characterized, and most of them showed down-regulation pattern under chilling stress. When transferred to 25°C for recovery (LT3), remarkable changes occurred in maize embryo. There were 873 DEGs including many PM related genes being identified between LT2 and LT3, some of which showing significant increase after 1 h recovery. Moreover, 15 genes encoding intracellular vesicular trafficking proteins were found to be exclusively differential expressed at recovery stage. It suggested that the intracellular vesicle trafficking might be essential for PM recovery through PM turnover. Furthermore, transcriptome analyses on imbibed embryos under normal condition (25°C) were also made as a contrast. A total of 651 DEGs were identified to mainly involved in protein metabolism, transcriptional regulation, signaling, and energy productions. Overall, the RNA-Seq results provided us a deep knowledge of imbibitional chilling injury on plasma membrane and a new view on PM repaired mechanism during early seed imbibition at transcriptional level. The DEGs identified in this work would be useful references in future seed germination research.

## Introduction

A rapid and proper seedling establishment after sowing plays a crucial role in maize (*Zea Mays* L.) productions. However, as one kind of thermophilic crops, maize is susceptible to chilling injury when sowed in early spring, inhibiting severely seed germination and seedling growth (Prasad, 1996). Thus, it is of great significance to study the physiological events during seed germination, especially under chilling conditions. Germination sensu stricto commences with the uptake of water (imbibition) and ends with the appearance of the embryonic axis through the surrounding structure (Nonogaki et al., 2010). It is a complex process referring to many kinds of metabolic reactions and signal transduction. Especially during seed early imbibition, the structural and functional recovery including resumption of energy metabolism, DNA/protein repair, activation of transcription and translation taken place severely (Weitbrecht et al., 2011). However, early imbibition is often accompanied by a massive leakage of cellular solutes, which is a sign of damage to membrane system derived from the desiccation (Leprince et al., 1993; Weitbrecht et al., 2011). Moerover, imbibition temperature makes deep affects on the severity of cellular leakage, which is called imbibitional chilling injury (Bewley et al., 2013). It has been well known that macromolecules and organelles often undergo a recovery process due to water absorption during early imbibition of dry seeds, such as the damages on proteins could be reversed by protein L-isoAsp-O-methyltransferase (PIMT) (Ogé et al., 2008) and DNA damages often were repaired by DNA ligases (Waterworth et al., 2010). However, the repair mechanism on damaged membrane was still unclear (Han and Yang, 2015).

The theory on membrane phase transition could be at present used for the explanation of imbibition injury. During seed maturation, the normal hydrophobic/hydrophilic orientation of membrane phospholipids is disrupted due to cell dehydration and the membrane changes to a less fluid gel state resulting in molecule movement restriction (Crowe et al., 1989). Once the rapid water absorption initiated at seed early imbibition, cellular damage, and leakage easily occurred due to the delayed membrane repairation to the normal liquid crystalline state (Bewley et al., 2013). And the imbibition damage is often severer under chilling condition. Moreover, a recent study on changes of membrane lipids during imbibition under normal and chilling temperatures in soybean seeds indicated that membrane lipid composition specifically reorganized during germination, and chilling injury was caused by PLD (phospholipase D)-mediated PA (phosphatidic acid) formation (Yu et al., 2015). However, how membrane recovered from impaired state, and what genes or proteins participated in this process still remained to be interpreted.

Membrane recovery played a prerequisite role for other cellular process during imbibition (Simon, 1974). Studies on plasma membrane (PM) repairing mechanism under abiotic stresses (such as drought, salinity, low temperature) indicated that PM resealing required intracellular vesicle trafficking including endocytosis and exocytosis (Togo et al., 1999; McNeil et al., 2003; Schapire et al., 2009; Los et al., 2011). The “patch hypothesis” explained that exocytosis triggered intracellular vesicles homotypic fusion to form a patch or reduced PM tension for resealing (McNeil and Kirchhausen, 2005). Endocytosis contributed to PM repair through removing lesions which caused by pore-forming proteins (Idone et al., 2008). Some regulatory and structural proteins involved in endomembrane trafficking had been well studied due to their potential role in PM repair (Battey et al., 1999; Sanderfoot and Raikhel, 1999; Pratelli et al., 2004). However, *Arabidopsis* Synaptotagmin 1 (SYT1) was up to now the only protein in plant demonstrated to participate in Ca^2+^-dependent repair of PM (Schapire et al., 2008; Yamazaki et al., 2008). Other components of PM repair identified in animals such as soluble N-ethymaleimide-sensitive factor attachment protein receptors (SNAREs) and annexins (Schapire et al., 2009) might have similar roles in plants, which still needed further study.

In this work, we try to illustrate the impacts of chilling imbibition on embryo plasma membrane at transcriptional level in maize seed, utilizing the prevalent RNA sequencing technology. The biological events occurred during seed germination had been well revealed by transcriptomic analysis in plants recently, such as *Arabidopsis* (Holdsworth et al., 2008; Kimura and Nambara, 2010), barley (Sreenivasulu et al., 2008), rice (Howell et al., 2009; Xu et al., 2012), sugar beet (Pestsova et al., 2008), and maize (Liu et al., 2013). However, little study was focus on seed early imbibition. In this study, the seeds of cold-sensitive maize inbred line Mo17 (Zheng et al., 2006) were used, and embryos were collected from dry seed (CK0), 2 HAI and 3 HAI (seeds imbibed under 25°C for 2–3 h respectively), LT2 (seeds imbibed at 5°C for 2 h) and LT3 (seeds imbibed at 5°C for 2 h and then transferred to 25°C for 1 h recovery). The results of RNA sequencing and subsequent analysis indicated that the expression of genes related to plasma membrane function changed remarkably. During the recovery period, it proposed that intracellular vesicle trafficking contributed to plasma membrane recovery via promoting plasma membrane turnover.

## Materials and methods

### Plant material

The chilling-sensitive maize (*Zea mays* L.) inbred Mo17 was used in this study. Dry seeds were surface sterilized with 0.5% NaClO for 5 min followed by thorough washing with water. Then seeds were subjected to normal and chilling imbibition respectively. Under normal condition (25°C), seeds imbibed between wet filter papers and were respectively sampled at 0, 2, and 3 h after imbibition (CK0, CK2, and CK3). Under chilling condition (5°C), seeds imbibed for 2 h (LT2) and then were transferred to 25°C for 1 h recovery (LT3). Embryos were rapidly separated from seeds of each sample, frozen in liquid nitrogen and stored at −80°C for RNA extraction.

### Measurements of physiological parameters

For malondialdehyde (MDA) content and antioxidant enzymes activities determination, about 0.1 g of embryos were ground in 5 ml of 0.05 M sodium phosphate buffer (pH 7.8) and centrifugated at 10,000 × g for 15 min. The supernatant was used for determining MDA concentration according to the method as described by Wang et al. (2013). Activities of catalase (CAT) and superoxide dismutase (SOD) were analyzed according to Pinhero et al. (1997).

### RNA isolation and transcriptome sequencing

Total RNA of each sample was extracted and purified using Quick Total RNA Isolation Kit (Waryong, Beijing, China). The quality and concentration of RNA was checked by Agilent Bioanalyzer 2100 system (Agilent Technologies, CA, USA) and NanoDrop system (Thermo Scientific, Wilmington, DE). The mazie RNA samples were sent to BGI (The Beijing Genomics Institute, China) for sequencing. The total RNA samples were digested with DNase I, and then the mRNA was enriched by using oligo (dT) magnetic beads and fragmented into short fragments. After double strand cDNA being synthesized by using random hexamer-primer, end reparation and 3′-end single nucleotide A (adenine) addition was performed. The fragments were enriched by PCR amplification for sequencing via Illumina HiSeq™ 4000. Raw sequence data are available in the NCBI's Short Read Archive (SRA) database with accession number SRP093477.

### Gene quantification and differential expression analysis

Generated clean reads were aligned to the reference gene ZmB73_5b_FGS_cdna (ftp://ftp.maizesequence.org/pub/maize/release-5b/filtered-set/ZmB73_5b_FGS_cdna.fasta.gz) and reference genome ZmB73RefGenv2 (ftp://ftp.maizesequence.org/pub/e/release-5b/assembly/masked/ZmB73_RefGen_v2.masked.maiztar.gz) using BWA (Li and Durbin, 2009) and Bowtie2 (Langmead et al., 2009) tools. Gene expression levels were estimated by RSEM for each sample. Clean data were mapped back onto the assembled transcriptome. Read count for each gene was obtained from the mapping results and normalized to FPKM (Fragments Per Kilobase of transcript per Million mapped reads). Screenings of DEGs are referred to the significance of digital gene expression profiles. Corrections for errors were performed using FDR (false discovery rate) method. The FDR ≤ 0.001 and absolute value of Log2 Ratio ≥ 1 were used as the default threshold to judge the significance of gene expression difference.

### Functional annotation

Gene Ontology (GO) enrichment analysis provides all GO terms that significantly enriched in lists of DEGs, comparing with a genome background, and filters the DEGs corresponding to specific biological functions. This method firstly mapped all DEGs to GO terms in the database (http://www.geneontology.org/), calculated gene numbers for every term and then used hypergeometric test to find significantly enriched GO terms based on GO Term Finder (http://www.yeastgenome.org/help/analyze/go-term-finder). KEGG (Kanehisa et al., 2008) (the major public pathway-related database) was used to identified significantly enriched metabolic pathways or signal transduction pathways in DEGs for further understanding genes biological functions. MapMan (Thimm et al., 2004) and PageMan (Usadel et al., 2009) analysis was employed to visualize metabolic overview of transcript changes between samples, based on fold changes of gene expressions (Log2 FC) and the mapping of Zm_B73_5b_FGS_cds_2012.

### Real-time quantitative PCR validation of RNA-Seq data

Total RNA of each sample was extracted and 500 ng of RNA were reverse-transcribed using PrimeScript™ RT reagent Kit (Takara, Dalian, China). Real-time RT PCR reaction was carried out using CFX96TM Real Time PCR Detection System (Bio-Rad, Hercules, CA, USA). Primer sets were designed with the Primer5 software and the maize actin gene was used as a control. Twenty microliter of reaction system contained 1 μl of diluted cDNA, 0.8 μl of reverse and forward primers, 7.4 μl of ddH_2_O and 10 μl of the AceQ qPCR SYBR Green Master Mix (Vazyme, Nanjing, China). Gene transcript abundance was calculated using the relative 2-ΔΔCT analytical method. Three biological replicates were conducted and each biological replicate was technically repeated three times. All data were expressed as the mean SD after normalization.

## Results

### Chilling induced membrane injury during seed early imbibition

**Figure 1:**
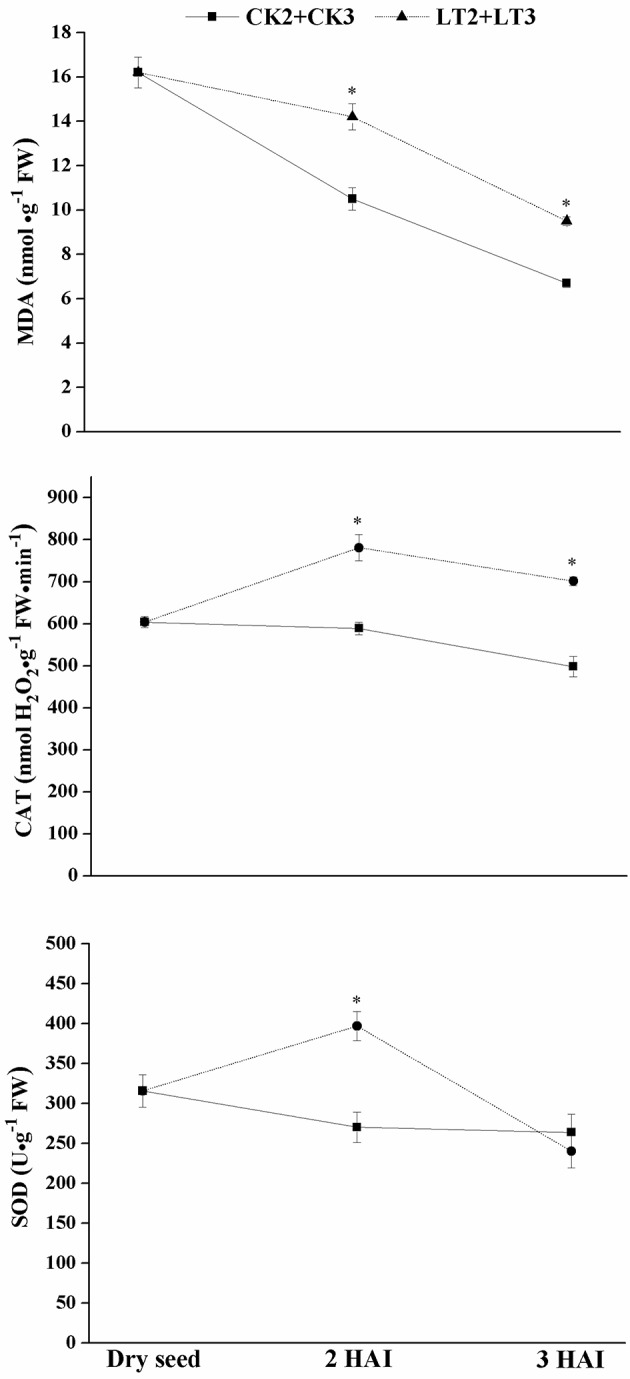
**Effect of chilling imbibition on MDA content and antioxidant enzymes activities in maize embryos**. Dry seed was used as CK0; 2 HAI and 3 HAI: maize seeds imbibed for 2–3 h in water; LT2+LT3: dry seeds were soaked in water for 2 h at 5°C (LT2), and then transferred to 25°C for 1 h recovery (LT3); CK2+CK3: dry seeds were soaked in water for 2 h at 25°C (CK2), and then continued imbibing at 25°C for 1 h (CK3); MDA: malondialdehyde, CAT: catalase, SOD: superoxide dismutase. The data were presented as the mean ± standard deviation of the mean (SD) and were tested for significant difference (*p* < 0.05, LSD).

The membrane disruption degree under chilling imbibition could be reflected by MDA content, a common product of lipid peroxidation. Under normal temperature, the highest MDA level was observed in dry seed, and then it decreased with increasing seed imbibition. The MDA content in chilling-imbibed embryo was significantly higher than that under normal temperature (Figure 1); while it declined significantly to the normal level at recovery stage. Besides, the activities of SOD and CAT increased obviously after 2 h chilling imbibition, but decreased at recovery period (Figure 1). The results indicated that low temperature inhibited normal recovery of cell membrane and even aggravated membrane damage.

### The overview of maize embryo transcriptome

Five RNA samples from maize embryos using RNA-Seq technology averagely generated 18,583,305 raw sequencing reads and 18,224,501 clean reads after filtering low quality. The clean reads were mapped to reference gene ZmB73_5b_FGS_cdna and reference genome ZmB73RefGenv2 using BWA/Bowtie2 tools. The average mapping ratio with reference gene/genome was 76.46 and 81.17% respectively. The summary of the sequencing data and alignment to reference for each sample was shown in Table 1.

**Table 1:** **Summary of the sequencing results during early imbibition of maize embryo**.

**Sample**	**Raw reads**	**Clean reads**	**Total mapped reads in gene (%)**	**Unique match in gene (%)**	**Total mapped reads in genome (%)**	**Unique match in genome (%)**
CK0^*^	18,634,821	18,371,393	74.09	57.53	81.62	65.82
CK2	15,125,186	14,837,739	77.69	60.21	80.65	68.62
CK3	21,343,712	20,872,802	77.24	59.99	80.52	68.21
LT2	20,107,393	19,648,277	77.90	60.18	80.78	68.82
LT3	17,705,417	17,392,295	75.37	58.00	82.27	66.74

* *Dry seeds were used as CK0; dry seeds were soaked in water for 2 h at 25°C (CK2), and continued imbibing at 25°C for 1 h (CK3); dry seeds were soaked in water for 2 h at 5°C (LT2), and then transferred to 25°C for 1 h recovery (LT3)*.

### Statistical analysis of differential expression genes

**Figure 2:**
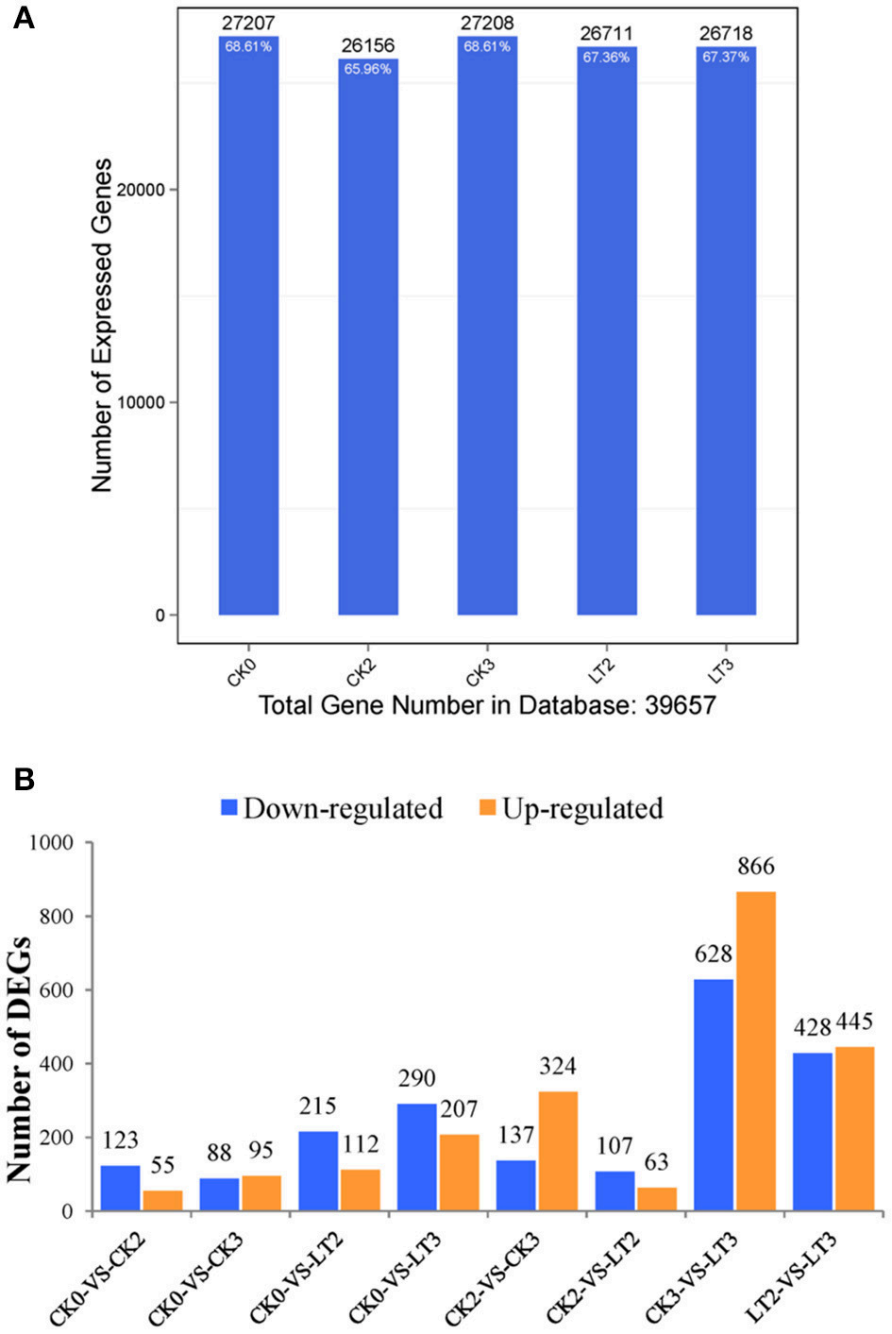
**Statistics of expressed genes during early imbibition of maize embryos. (A)** the number of detected expressed genes and its proportion to total gene number in database for each sample. **(B)** the number of up-regulated and down-regulated DEGs (differentially expressed genes) in each pairwise comparison. Other explanations see Figure 1.

Gene expression level was quantified by FPKM (Fragments Per Kilobase of transcript per Million mapped reads) method. The number of genes identified in each imbibed embryo sample was approximately 27,000, accounting for 67% of total genes in maize database (Figure 2A). It suggested that little change happened in embryo transcriptome during early imbibition of maize. Eight comparisons from five samples were made and the number of DEGs was shown in Figure 2B (all detected DEGs of each comparison were listed in Supplementary Files 1–8). Only 178 DEGs were identified at CK2; while 327 DEGs at LT2. In addition, a higher proportion of genes were down-regulated during chilling imbibition. From 2 HAI to 3 HAI, dramatic changes occurred in imbibed embryo, reflected by 461 DEGs (CK2 vs. CK3) and 873 DEGs (LT2 vs. LT3), and more genes were up-regulated. However, as comparing with dry seed, transcriptomes of 3 HAI (CK0 vs. CK3, CK0 vs. LT3) just changed a little. It indicated a dynamic profile due to decomposition and synthesis at transcription level in early imbibition embryo.

### Functional annotation

**Figure 3:**
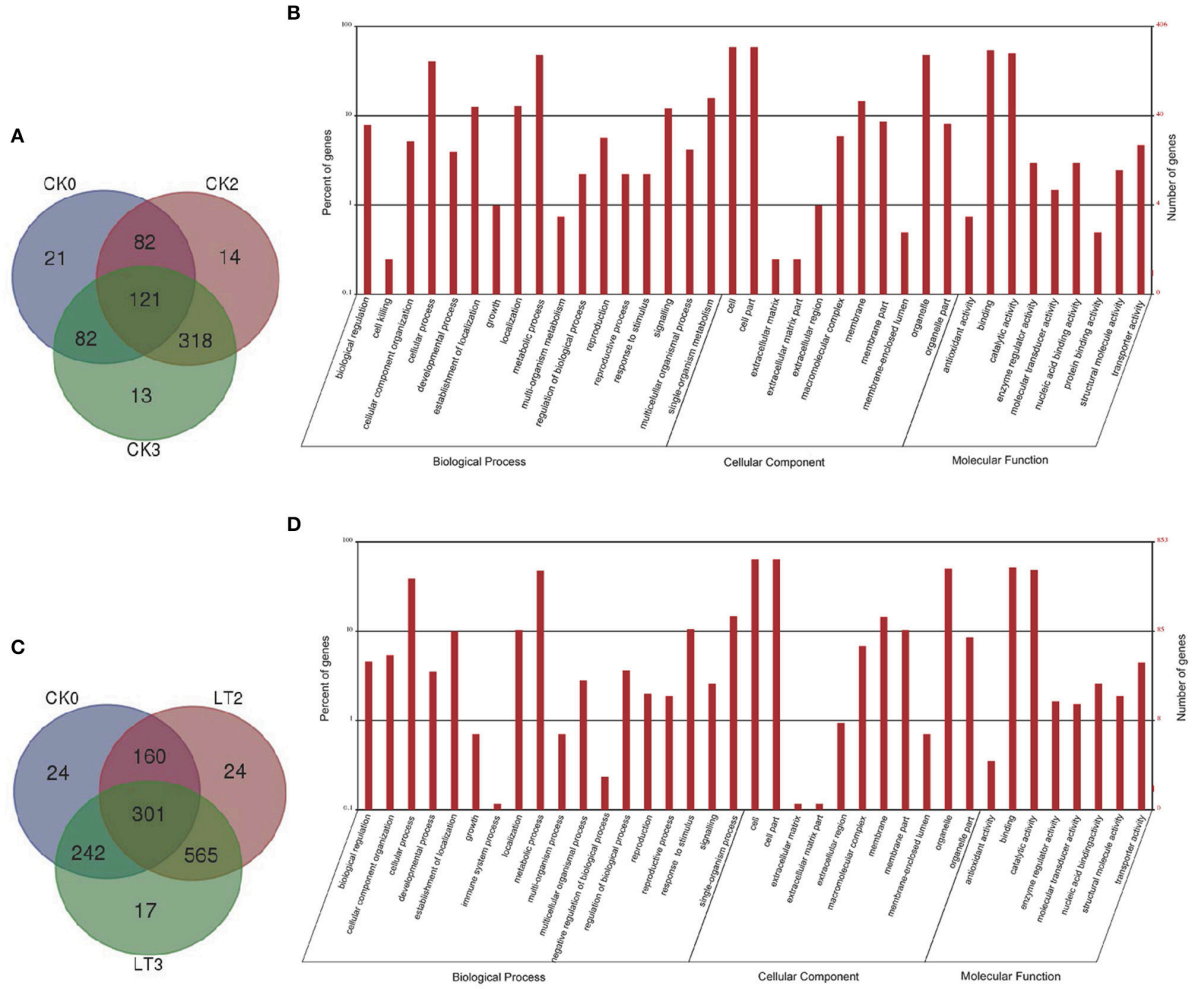
**Differentially expressed genes (DEGs) and their Gene Ontology (GO) annotations during early imbibition of maize embryos. (A)** venn diagram of DEGs distributed in maize embryo samples during seed imbibition at 25°C; **(B)** histogram of GO classifications of the DEGs in maize embryos under 25°C imbibition. Total 406 DEGs were annotated to three main categories. The y-axis on the right side indicated the number of genes and y-axis on the left side meant the percent of genes in a category; **(C)** venn diagram of DEGs distributed in maize embryo samples under chilling condition; **(D)** histogram of GO classifications of the DEGs in maize embryos under 5°–25°C imbibition. Total 853 DEGs were annotated. Other explanations see Figure 1.

The annotation analyses of Gene Ontology (GO) were performed on screened DEGs of each comparison (Supplementary Files 1–8). GO functional classification was used to detect the distribution of gene functions of the specie from the macro level. Among 651 DEGs in normal imbibed embryos, 406 were assigned into different GO terms; while 853 of 1333 DEGs detected under chilling imbibition could be annotated. GO annotations under both conditions had similar results (Figure 3): in biological process category, several metabolic processes like localization, signaling, response to stimulus enriched obviously; membrane term occupied a major part in cellular component; most genes related to binding and catalytic activities as referred to molecular function. Genes usually interacted with each other to play roles in certain biological functions. The pathway enrichment analysis of DEGs based on KEGG database was performed and a scatter plot for the top 20 of KEGG enrichment results in eight comparisons were shown in Supplementary Figure 1.

### Differential expression of maize embryo genes during early imbibition

**Figure 4:**
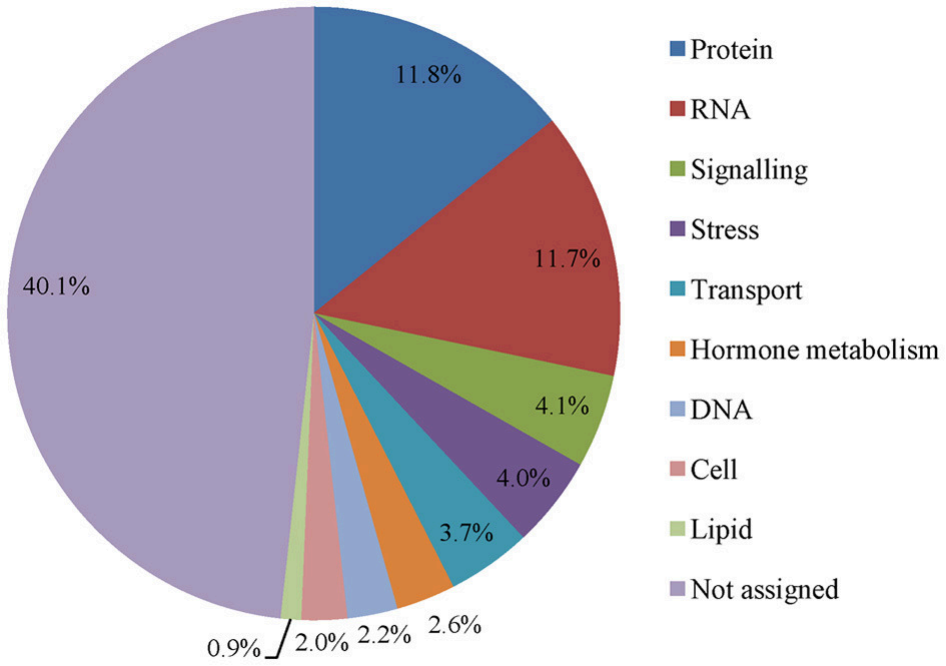
**Functional distributions of transcripts in early imbibed maize embryos at 25°C**. Functional categories were obtained from Mapman software. The proportion of each category was calculated.

MAPMAN analysis was used to make functional categories for maize embryo transcriptomes during early imbibition. Total DEGs of CK0, CK2, and CK3 were collected and the proportion of each category was calculated (Figure 4). Protein and RNA were top two functional categories with the greatest number of transcripts, indicating the most importance, and priority of protein and RNA metabolism upon imbibition. Other important groups were related to signaling, stress, transport and so on. Besides, 40% genes belonged to unknown or unassigned function category.

**Figure 5:**
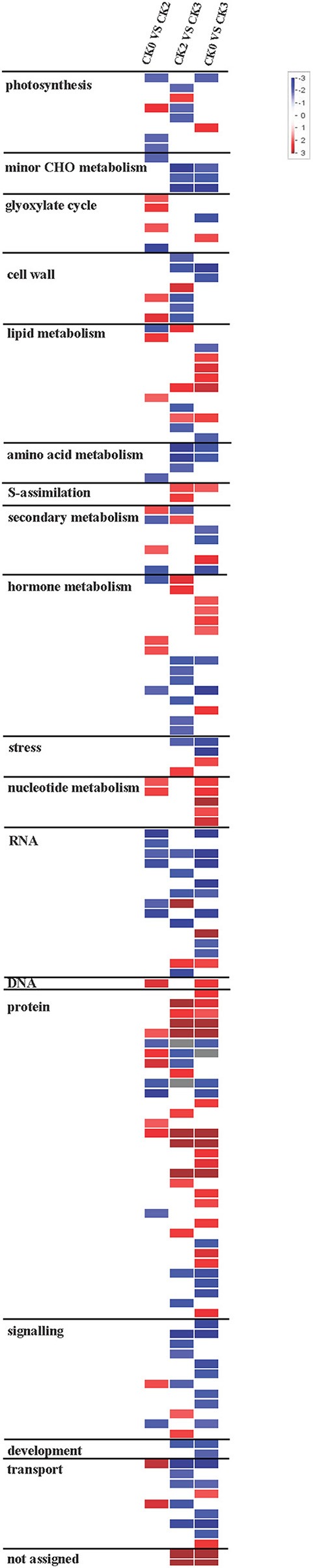
**Enriched categories of differentially expressed genes during early imbibition of maize embryos**. Each column represents the genes that are significantly up (red) or down (blue) regulated between two samples. Functional categories were obtained from PageMan software. Other explanations see Figure 1.

The expression profile of enriched functional categories in three pairwise comparisons was obtained by PageMan analysis and Wilcoxon test (Figure 5). Most of enriched categories showed a down-regulated pattern, including cell wall, RNA metabolism, signaling, development and transport. Interestingly, genes encoding protein metabolism like amino acid activation and protein synthesis were greatly up regulated at three comparisons; while genes encoding RNA metabolism especially transcription factors were nearly all down regulated.

#### RNA metabolism

**Figure 6:**
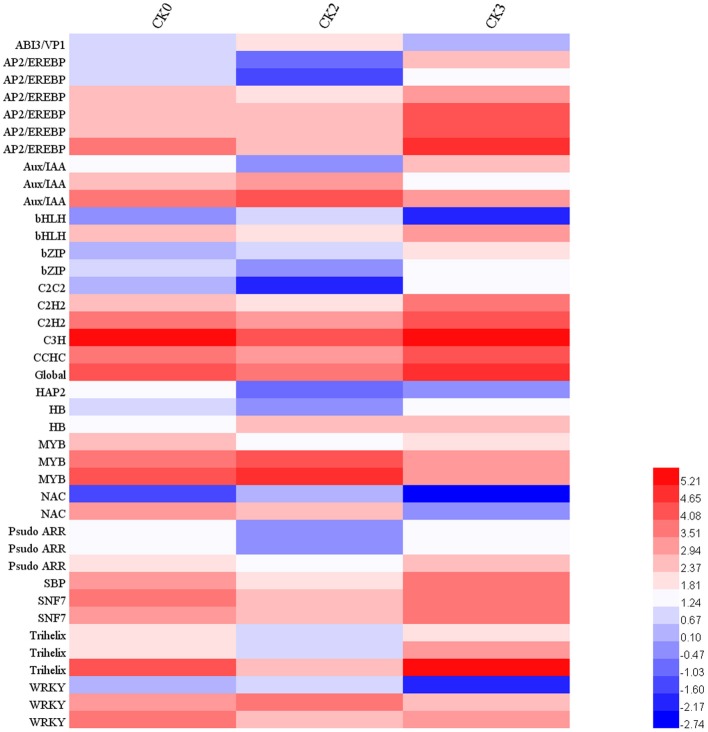
**Expression profiles of transcription factors during early imbibition of maize embryos**. Identification of transcription factors was derived from MapMan annotations. Forty one mRNAs encoding transcription factor were characterized, which belong to 18 TF families. Their expression profiles were illustrated by Log2 transformation of absolute abundance. Other explanations see Figure 1.

Genes assigned to the RNA category comprised a large proportion of embryo transcriptome during early imbibition (Figure 4) and almost all the transcripts involving in RNA processing and regulations were down-regulated (Figure 5), implying a suppressed transcription activity at this stage. In addition, given that most of transcripts in RNA category had been assigned to different transcription factor families, a further analysis on transcription factors during seed germination were carried out. As is shown in Figure 6, 41 mRNAs encoding transcription factors (TF) were characterized among DEGs under normal imbibition (Figure 6), and TF families included 18 members such as AP2/EREBP, Aux/IAA, bHLH, and WRKY showed potential roles in seed germination of rice (Howell et al., 2009) and barley (Sreenivasulu et al., 2008).

#### Protein metabolism

Protein synthesis was required for seed germination (Rajjou et al., 2004). The genes involved in protein synthesis were nearly all up-regulated in this study, including amino acid activation, and ribosomal proteins based on MAPMAN category (Figure 5). Meanwhile, genes participating in protein degradation, such as cysteine protease, serine protease, aspartate protease, and especially ubiquitin showed changed expression patterns. Most of transcripts assigned to RNA metabolism encoded protein phosphatases, which belonging to post-translational modification proteins, suggesting a molecular control of seed germination at posttranslational level.

### Transcriptome analysis of imbibitional chilling injury on plasma membrane

In order to illustrate the influence of chilling on imbibed embryo, transcriptional changes analysis between CK0 and LT2 was carried out. Based on GO cellular component term, functional annotations by MAPMAN and previous studies on homolog of identified DEGs in *Arabidopsis* and rice. Among 327 DEGs indentified between CK0 and LT2 (Supplementary File 4), 15 specific genes with PM relevant functions belonging to lipid metabolism, stress, signaling, and transport categories were characterized, and most of them showed down-regulation pattern under chilling stress (Table 2). The results indicated that chilling imbibition negatively affected PM function, leading to the decrease of membrane stability, signal transduction, and transport metabolic activity.

**Table 2:** **The differentially expressed genes with PM-related function during chilling imbibition of maize embryo**.

**Gene ID**	**Log2 ratio**	**Encoding genes**
*LIPID METABOLISM*		
GRMZM2G316362	−5.0	Putative stearoyl-ACP desaturase
GRMZM2G107839	−1.6	Putative lipid transfer protein 3
*STRESS. COLD*		
GRMZM2G071292	−6.8	C2 domain-containing protein
*SIGNALING*		
GRMZM2G422340	−1.8	Putative S-locus receptor-like protein kinase family protein, similar to ZmPK1 precusor
AC233871.1_FG003	1.7	Putative calcium-dependent protein kinase 19
GRMZM2G398341	−7.3	Putative phosphatidylinositol 4-OH kinase beta1(PI4K β1)
GRMZM2G332410	7.0	Putative PI4K β1
GRMZM2G112247	−1.8	Similar to SEC14 protein
GRMZM2G025579	−2.5	Hypothetical protein, simiar to *Arabidopsis* histidine kinase 5(AHK5)
*VESICLE TRANSPORT*		
GRMZM2G022188	−3.0	Putative coatomer beta subunit family protein
GRMZM2G085295	−1.9	clathrin heavy chain protein
*TRANSPORT*		
GRMZM2G326677	−1.2	Similar Aminophospholipid ATPase3
AC199911.5_FG001	−1.7	Hypothetical protein, similar to V-type H^+^-transporting ATPase subunit D
GRMZM2G104942	−3.2	Putative glycerol-3-phosphate transporter
GRMZM2G161168	−1.9	Putative transport protein in plasma membrane

### Plasma membrane recovery from imbibitional chilling injury

After 2 h of chilling imbibition and 1 h recovery at 25°C, transcriptome comparative analysis on LT2 and LT3 was carried out (Supplementary File 5). Eight Hundred Seventy Three DEGs were identified, which included many PM related genes (Supplementary Table 1), such as fatty acid synthesis and elongation, cold stress, signaling, cell organization, and transport. Several transcripts which were down-regulated upon chilling imbibition, such as ACP desaturase, signaling proteins and ATPases, showed significant increase in abundance after 1 h recovery, implying a recovery of PM activity under normal temperature.

### Transcripts relevant to vesicle transport during recovery period

The exclusively differential expressed transcripts related to vesicle transport at recovery stage were found after bidirectional transcriptomic comparisons of LT2 vs. LT3 and CK3 vs. LT3 (Table 3). They mainly encoded coatomer, clathrin and SNAREs, which constituted basic vesicle transport proteins. Vesicular trafficking was essential for plant development. Its role in plasma membrane turnover was fundamental for intracellular vesicle transport (Steer, 1988). Genes encoding vesicle transport proteins were detected for exclusively differential expression during the recovery period, indicating that the intracellular vesicle trafficking was essential for plasma membrane recovery through PM turnover.

**Table 3:** **Differently expressed transcripts relevant to vesicle transport during recovery period of maize embryo**.

**Gene ID**	**Description**
GRMZM2G111611	Vesicle transport v-SNARE 13
GRMZM2G318459	Hypothetical protein, similar to VAP (VESICLE ASSOCIATED PROTEIN) 27 in rice
GRMZM5G817310	May encode COPI (coatomer protein I)
GRMZM2G065884	May encode COPI (coatomer protein I)
GRMZM2G438895	Hypothetical protein, similar to chaperone DnaJ-domain superfamily protein
GRMZM2G075496	Oxysterol-binding protein OBPa
GRMZM2G085295	Clathrin heavy chain
AC155622.2_FG001	Hypothetical protein, similar to coatomer alpha subunit
GRMZM2G149406	Hypothetical protein, similar to coatomer alpha subunit
GRMZM2G316635	Hypothetical protein, similar to chaperone DnaJ-domain superfamily protein
GRMZM2G336789	TPA: AP-1 complex subunit sigma-2
GRMZM2G350795	Hypothetical protein, similar to novel plant SNARE 11
GRMZM2G003124	Syntaxin 81
GRMZM2G010054	Hypothetical protein, similar to coatomer subunit alpha
GRMZM2G390489	Unknown protein

### Real-time quantitative PCR validation of RNA-Seq results

**Figure 7:**
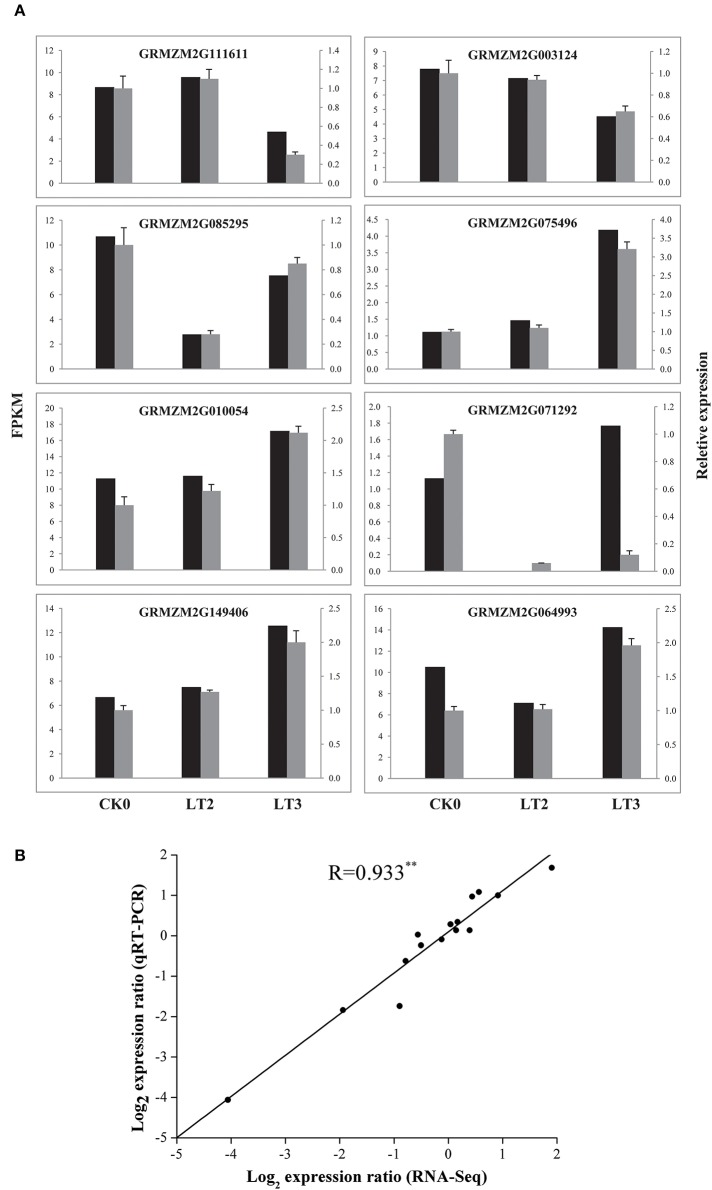
**The real-time quantitative PCR (qRT-PCR) validation of RNA-Seq results. (A)** qRT-PCR verification of vesicle trafficking related genes during recovery process. Black column represented the FPKM of a certain gene; while gray column indicated its relative expression levels analyzed by qRT-PCR. The relative mRNA levels were normalized with reference gene (actin). The genes and primer sequences used for qRT-PCR analysis are shown in Supplementary Table 2. **(B)** Pearson correlation analysis of gene expression ratios obtained from the qRT-PCR and RNA-Seq data. The qRT-PCR log2 values (ratios of LT2/T1 and LT3/T1, y-axis) were plotted against the RNA-Seq log2 values (FPKM ratios of LT2/T1 and LT3/T1, x-axis). The Pearson correlation coefficient (R) was given in the plot indicated the extremely significant difference at *P* < 0.01. Other explanations see Figure 1.

Expression patterns of eight selected genes involving in vesicle trafficking were analyzed through qRT-PCR assays. Comparison of qRT-PCR and RNA-Seq data showed consistent expression trends in all eight genes. Besides, a Pearson correlation analysis between the gene expression levels measured by qRT-PCR and RNA-Seq showed a highly significant correlation (correlation coefficient *R* = 0.933, *P* < 0.01), supporting the reliability of sequencing results (Figure 7).

## Discussion

### Impact of chilling imbibition on plasma membrane at transcript level

The results from MDA and antioxidant enzymes measurements indicated that the injury induced by chilling on membrane of imbibed embryo could be alleviated after recovery under normal condition. Subsequent transcriptome analysis further revealed that chilling imbibition made adverse effect on plasma membrane, which reflected by the universal down-regulation of genes encoding PM related proteins. Chilling decreased the plasma membrane fluidity, leading to the fatty acid unsaturation which was catalyzed by lipid desaturases (Wang et al., 2006; Upchurch, 2008; Barkla and Pantoja, 2011). GRMZM2G316362 was found encoding a putative stearoyl-ACP desaturase (SAD) in this work, which was an important member of fatty acid (FA) desaturase family with function of catalyzing the first fatty acids desaturation step in plastid (Thelen and Ohlrogge, 2002). It has been proposed low temperature induced transcription of desaturase in maize (Madi et al., 2003). Another lipid metabolism related transcript (GRMZM2G107839) encoding a putative lipid transfer protein 3 decreased in abundance. A research on maize lipid transfer protein 3 (ZmLTP3) showed a role in maintaining the stability of PM under salt tress and thus minimize dehydration in living cells (Zou et al., 2013). Further, these two genes were also deferentially expressed and down regulated in CK2-LT2 comparison (Supplementary File 7). It indicated that chilling easily caused alterations on embryo PM during early imbibition in terms of lipid unsaturation and membrane stability.

PM played a central role in the regulation network of plant responding to abiotic environmental conditions, because it was the site of sensors that perceived and transduced environment signal to intracellular part (Takahashi et al., 2013). The proteomics research on *Arabidopsis* plasma membrane identified 238 proteins, in which 38 proteins (16%) including receptor-like protein kinases (RLKs) and calcium-dependent protein kinases (CDPKs) involved in signal transduction (Alexandersson et al., 2004). Genes encoding similar function proteins were also identified in our study. GRMZM2G422340 encoding a putative receptor-like protein kinase (RLK) with the self-incompatibility locus (S-locus) domain was down regulated and similar with ZmPK1, which was the first plant RLK gene isolated form plasma membranes of young maize seedlings (Walker and Zhang, 1990). CDPK19 was a member of calcium-dependent protein kinase (CDPK) family. Its homolog in *Arabidopsis* (At5g19450) showed PM localization pattern regulating stomata movement (Hubbard et al., 2011), however the physiological function of CDPK19 in maize was still unknown. Two transcripts encoded same putative protein kinase PI4K β1 (phosphatidylinositol 4-OH kinase beta1), which in *Arabidopsis* was known as a membrane regulator of endocytosis (Fan et al., 2015). Thus, its differential expressions in maize embryo suggested possible involvement in endocytosis pathway during chilling imbibition. Other membrane related transcripts characterized in this work were also supposed to participate in signaling pathways in response to various stresses (Iwama et al., 2007; Mousley et al., 2007; Desikan et al., 2008; Mira-Rodado et al., 2012; Pham and Desikan, 2012). Above all, several signaling related genes encoding putative kinase proteins were identified during chilling imbibition in maize embryo; however, none of them obtained studied to our knowledge until now.

The PM transport and metabolic activity easily suffered from the effect of low temperature (Ishikawa and Yoshida, 1985; Peng et al., 2008). In accordance with these findings, genes encoding two ATPases, a glycerol-3-phosphate transporter and an unknown PM transporter, were detected under chilling imbibition. Activity reduction or inactivation of ATPases on the plasma membrane decreased cell absorption and transportation function, and also broken the balance of materials exchange between plants and the surrounding environment.

### Plasma membrane recovery from imbibitional chilling injury

Many PM related genes with functions of signaling and cell organization were identified in transcriptome analysis of embryo during recovery period. Receptor protein kinases (RPKs) were important mediators in response to stimulus in plant cell and many of them were integral plasma membrane proteins (Becraft, 2002). The study on global profile of gene expression induced by low temperature in rice found out several genes encoding plasma membrane RPKs (Chawade et al., 2013). Similarly, 11 genes were identified to encode RPKs in our study, containing several typical RPKs families such as leucine rich repeat (LRR), receptor like kinase1 (RLK1), DUF26 and wall associated kinase (WAK). In addition, some genes relevant to cell organization showed high similarities to tubulin family. GRMZM2G334899 encoded tubulin beta-2 chain in maize, which was similar to tub6 in *Arabidopsis*. Tub6 was a PM localization member of β tubulin, which was the main assembling component of microtubules to form the cytoskeleton. Recently, microtubules as sensors to abiotic stimuli (salt, drought, and low temperature) had drawn many attentions. They were not only the action site of cold stress, but also participated in cold sensing as modulators of calcium-channel activity (Nick, 2008). It was proposed that the differentially expressed tubulin genes might play dual functions during recovery period, promoting the structural reorganization inside the cell, as well as participating in signal transduction pathway.

### Vesicle transport participated in plasma membrane turnover

The intracellular vesicular transports including exocytosis and endocytosis were fundamental features of the eukaryotic cells (Robinson et al., 1998). They acted in the transfer of transmembrane proteins or lipids between endomembrane system and plasma membrane. The secretion and endocytosis in plant cells were facilitated by vesicles, many of which were “coated” on the cytoplasmic surface. Researches on mammalian and yeast cells had discovered different types of coated vesicles, including clathrin coated vesicle with adaptor complexes, coatomer protein I and coatomer protein II (Hwang and Robinson, 2009). While in plant cells, more isoforms of coat proteins like N-ethylmaleimide-sensitive factor adaptor protein receptors (SNAREs) were found out, which were key players in vesicle-associated membrane fusion events (Lipka et al., 2007). Vesicular trafficking was essential for various aspects of plant development and signal transduction, including gravitropism, cytokinesis, and stress responses (Surpin and Raikhel, 2004). Its role in plasma membrane turnover in plant cells drew our more attention due to its fundamental role in intracellular vesicle transport. Plasma membrane turnover in plant cells required the coordination of exocytosis and endocytosis: exocytosis incorporated new membrane into cell surface, while endocytosis internalized surface membrane to create new plasma membrane (Steer, 1988). Researches on exocytosis and endocytosis were mainly carried on materials from root hair tips (Ovečka et al., 2005) or growing pollen tubes (Andrés and Du, 2011) because of their high endosomal trafficking activities. In our study, maize radical tips were used and several genes encoding vesicle transport were detected differentially expressed during recovery period from chilling imbibition. It suggested that intracellular vesicle transport might participate in plasma membrane recovery through PM turnover process.

### Plasma membrane repair involved in membrane recovery

Three transcripts GRMZM2G064993, GRMZM2G071292, and AC210204.3_FG002 were highlighted because of their possible role in plasma membrane repair. GRMZM2G064993 encoded annexin p33 protein in maize. Annexins were widely expressed multigene family of Ca^2+^-dependent, phospholipid-binding proteins in various plants, animals and fungi. They played important roles in plant growth and development (Mortimer et al., 2008; Laohavisit and Davies, 2009; Konopka-Postupolska et al., 2011; Clark et al., 2012). Although the functions of annexins in plant cells on PM repair had not been proposed, their counterparts were found to be essential in PM resealing in animal cells (Lennon et al., 2003; McNeil et al., 2006). In maize, only two annexin genes (*ZmAnn33* and *ZmAnn35*) have been characterized and the functions remain to be determined (Battey et al., 1996). Annexin proteins ZmAnn33/35 had been reported to create directly Ca^2+^-permeable transport pathways (Laohavisit et al., 2009). Therefore, the characterization of *ZmAnn33* during recovery from chilling injury in our study implied its possible PM repair function. Another two transcripts GRMZM2G071292 and AC210204.3_FG002 encoding C2 domain-containing proteins were found to response to cold stress. C2 domains were known as phospholipid and Ca^2+^-binding domains, which had been found in numerous signaling proteins interacting with cellular membranes (Nalefski and Falke, 1996). Synaptotagmins and dysferlin containing multiple copies of C2 domains were two important components in PM repair identified in animal cells (Reddy et al., 2001; Südhof, 2002; Bansal and Campbell, 2004). Thus, it was proposed that other proteins with C2 domains such as SYT1 homolog might have the similar roles in PM repair in plant cells (Schapire et al., 2009). Because of the membrane binding property conferred by C2 domain, these two identified transcripts probably participated in membrane recovery process in maize embryo after chilling stress.

**Figure 8:**
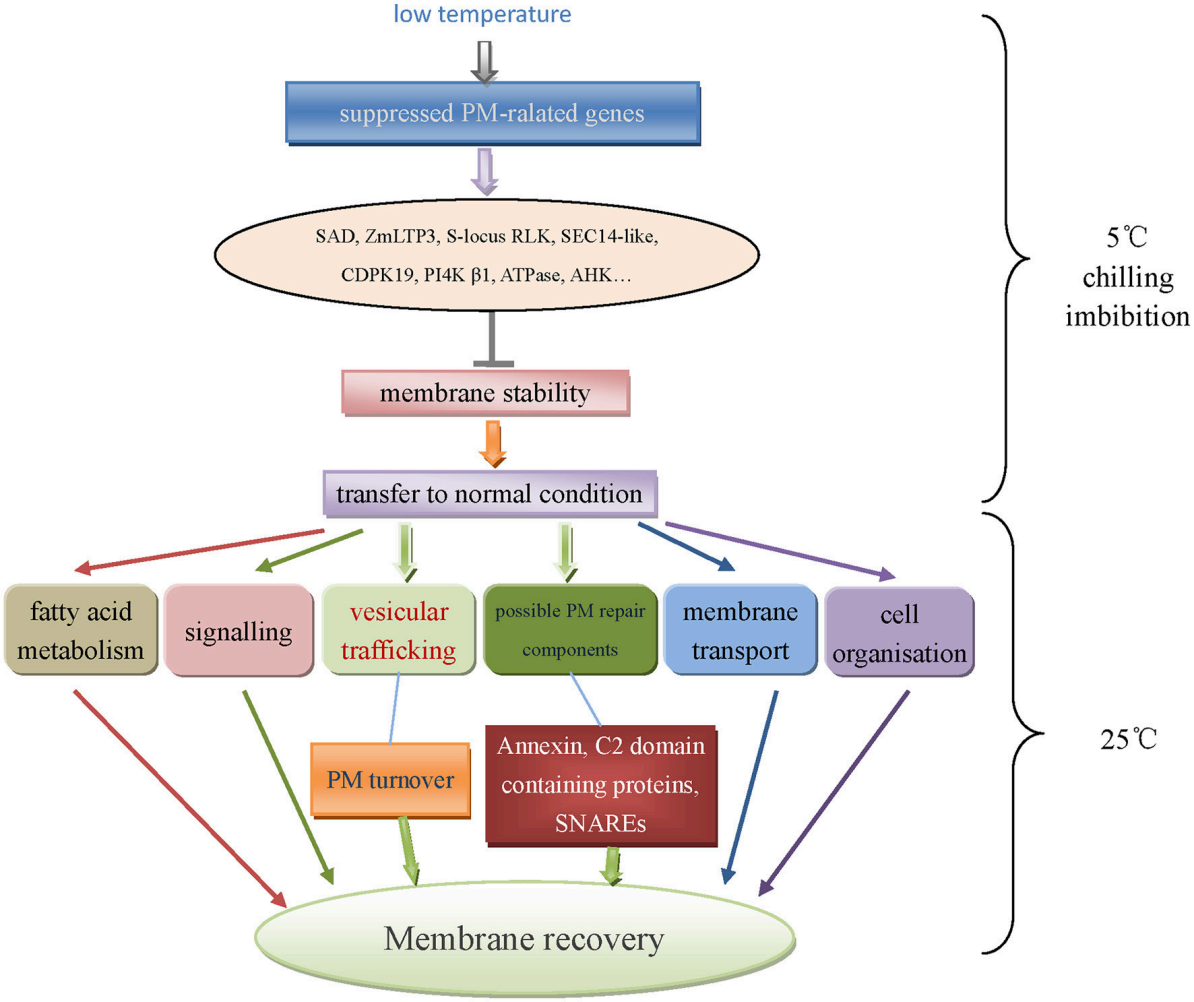
**A possible network of plasma membrane responding to low temperature during chilling imbibition and recovery period of maize embryo**.

Finally, a network of plasma membrane responding to low temperature during chilling imbibition and recovery period in maize embryos was illustrated in Figure 8. In summary, low temperature negatively affected the PM normal functions (signaling, transport etc.), however the embryo membrane would undergo a recovery process when transferred to normal condition. The transcripts down-regulated upon chilling imbibition significantly increased during recovery. It was proposed that PM recovery was facilitated by intracellular vesicle trafficking through PM turnover process. Besides, potential PM repair components such as annexins or C2 domain-containing proteins might play important roles in this process.

## Conclusions

In this study, we conducted transcriptomic analysis by RNA-seq of embryos at early stages of imbibition under normal and chilling conditions in maize. Transcriptome of normal imbibed embryos showed little changing pattern, reflected by small amount of DEGs identified during this period. It indicated that weak physiological activities occurred at very early imbibition. Genes differentially expressed at early imbibition were supposed to participate in fundamental metabolic process prior to seed germination. Further analysis assigned theses genes to functional categories mainly consisting of transcriptional factors regulation, protein synthesis and degradation, signaling, transport etc.

Besides, maize seeds were subjected to 5°C for 2 h imbibition to induce chilling injury to embryos. That the PM relevant genes were universally down-regulated in transcriptome analysis suggested that low temperature negatively affected PM function, including lipid metabolism, signaling and transport activity. Chilling imbibed seeds were then placed under 25°C for 1 h recovery. Comparative transcriptome analysis identified genes mainly with functions of fatty acid synthesis and elongation, cold stress, signaling, cell organization, and transport. The possible role of vesicle transport was highlighted in this study, and it was proposed that plasma membrane turnover process driven by intracellular vesicle trafficking could promote PM recovery from imbibitional chilling injury, which might be a new point of view for further study on membrane recovery during seed germination. The transcriptome data provided valuable information of genes expressed at early seed imbibition, while their further biological functions still remained to be discovered.

## Author contributions

FH designed and performed the experiments, analyzed the data, and finally wrote the paper. HS performed the experiments, analyzed the data, and wrote the paper. CL designed the experiments and wrote the paper. HF performed the experiments and wrote the paper. MS performed the experiments and analyzed the data. YG conceived and designed the experiments, wrote and revised the paper. YH performed the real-time PCR. JH revised the paper.

## Funding

This research was supported by the National Natural Science Foundation of China (No. 31201279, 31371708, 31671774), Special Fund for Agro-scientific Research in the Public Interest (No. 201203052), Zhejiang Provincial Natural Science Foundation (No.LY15C130002, LZ14C130002) and Jiangsu Collaborative Innovation Center for Modern Crop Production, P. R. China.

### Conflict of interest statement

The authors declare that the research was conducted in the absence of any commercial or financial relationships that could be construed as a potential conflict of interest.
