# Supplementary figures and images for: Transcriptome Analysis of Chilling-Imbibed Embryo Revealed Membrane Recovery Related Genes in Maize
Source: Front Plant Sci. 2017 Jan 4;7:1978. doi: 10.3389/fpls.2016.01978 (PMC5209358; doi:10.3389/fpls.2016.01978)

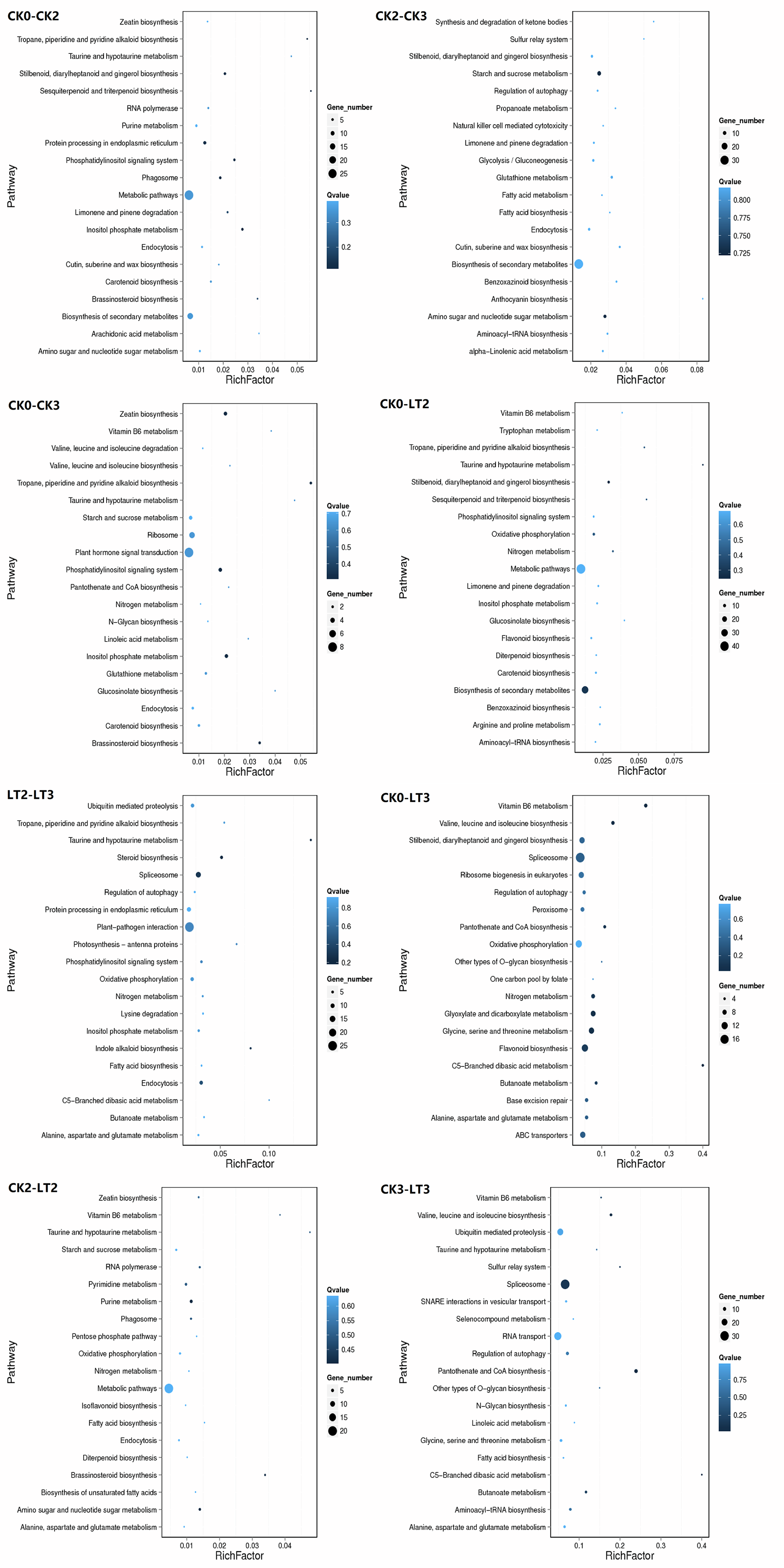

Supplement: Supplementary file 9 [file Image1.TIF]
