# Supplementary material for: Transcriptome Analysis of Chilling-Imbibed Embryo Revealed Membrane Recovery Related Genes in Maize
Source: Front Plant Sci. 2017 Jan 4;7:1978. doi: 10.3389/fpls.2016.01978 (PMC5209358; doi:10.3389/fpls.2016.01978)
Supplement: Supplementary file 10 [file Presentation1.PDF]

Supplementary Materials

Title: Transcriptome analysis of chilling-imbibed embryo revealed membrane recovery related genes in maize

Fei He, Hangqi Shen, Cheng Lin, Hong Fu, Mohamed Salah Sheteiwy, Yajing Guan*, Yutao Huang, Jin Hu*

*** Correspondence:** Corresponding Author: Yajing Guan, E-mail: vcguan@zju.edu.cn; Jin Hu, E-mail: jhu@zju.edu.cn

1. Supplementary Data Sheet

Supplementary File 1 to 8 contain differential expression genes (DEGs) identified in each comparison.

2. Supplementary Figures

Supplementary Figure 1. Statistics of pathway enrichment of DEGs in each pairwise during early imbibition of maize embryos. RichFactor was the ratio of differentially expressed gene numbers annotated to all gene numbers annotated in one pathway. Greater richFator meant greater intensiveness. Q-value was corrected p-value ranging from 0-1, and less Q-value meant greater intensiveness. The top 20 of enriched pathway terms were displayed in each pairwise. Other explanations see Figure 1.

3 Supplementary Tables

Supplementary Table 1. The differentially expressed genes with PM-related function during recovery period of maize embryo.

Gene ID	Log2 ratio	Encoding genes
Lipid metabolism		
GRMZM2G062357	1.3	Putative acyl-activating enzyme 18
GRMZM2G176546	1.3	Putative long-chain acyl-CoA synthetase 6
GRMZM2G026793	1	Highly similar to acyl-ACP desaturase in rice
AC215690.3_FG002	1.2	Highly similar to acyl-ACP desaturase in rice
GRMZM2G112942	-3.6	Putative GLTP2 (glycolipid transfer protein 2)
GRMZM2G087275	-1.4	Highly similar to serine palmitoyltransferase 1 in rice
Stress. cold		
GRMZM2G071292	7.5	C2 domain-containing protein
AC210204.3_FG002	2.5	C2 domain-containing protein
Signaling		

GRMZM2G150448	1.3	Putative leucine-rich repeat receptor-like protein kinase family protein (Zea mays)
GRMZM2G045981	1.2	Putative leucine-rich repeat receptor-like protein kinase family protein (Zea mays)
GRMZM2G161452	-1.1	Protein kinase family protein
GRMZM2G149051	1.5	TPA: putative leucine-rich repeat receptor protein kinase family protein (Zea mays)
GRMZM2G072569	1.3	TPA: putative leucine-rich repeat receptor protein kinase family protein (Zea mays)
GRMZM2G389948	-1.1	Putative leucine-rich repeat receptor-like protein kinase family protein (Zea mays)
GRMZM2G006080	1.3	Putative leucine-rich repeat receptor-like protein kinase family protein (Zea mays)
GRMZM2G100288	1.2	TPA: putative leucine-rich repeat receptor protein kinase family protein (Zea mays)
GRMZM2G027821	1.3	DUF614 containing protein
GRMZM2G013790	-1.5	LRK10 like protein
GRMZM2G305211	-1.3	Similar to 14-3-3-like protein in rice
GRMZM2G097856	-1.1	Similar to 14-3-3-like protein in rice
GRMZM2G025579	2.5	Nearly identical to AHK5 (arabidopsis histidine kinase 5) in rice
GRMZM2G013318	1.2	Putative GTP-binding protein
GRMZM2G156986	1.1	Putative GTP-binding protein
Cell organization		
GRMZM2G338333	1.2	Highly similar to ITN1 (increased tolerance to nacl) in arabidopsis
GRMZM2G064993	1	Annexin-like protein RJ4
GRMZM2G334899	3	Tubulin beta-2 chain (Zea mays)
GRMZM2G020982	1.1	Highly similar to ITN1 (increased tolerance to nacl) in arabidopsis
GRMZM2G136996	1.1	Protein binding protein (Zea mays), highly similar to ankyrin repeat family protein
Transport		
GRMZM2G467992	-1	Putative ATP synthase subunit H family protein
GRMZM2G019404	-1.2	TPA: plasma-membrane H^+ATPase2 isoform 1 (Zea mays)
GRMZM2G072850	1.3	Nearly identical to multidrug resistance protein 11 in rice
GRMZM2G060045	1.9	Nearly identical to peroxisomal abc transporter 1 in arabidopsis
GRMZM2G125424	1	Nearly identical to multidrug resistance protein 11 in rice
GRMZM2G113203	1.7	Multidrug resistance protein associated1 [Zea mays]
GRMZM2G479906	1.9	Putative pleiotropic drug resistance protein 4
AC194112.3_FG001	-1.2	Putative ABC transporter family protein
GRMZM2G035276	-1.2	Putative ABC transporter family protein
GRMZM2G011592	1.1	Putative Ca2+cation exchanger

GRMZM2G017388	1.3	Putative Ca2+cation exchanger
GRMZM2G104942	4.1	Putative glycerol-3-phosphate transporter
GRMZM5G843302	1	Putative glycerol-3-phosphate transporter

Supplementary Table 2. Primer sequences of genes used for qRT-PCR analysis

**Gene ID**	**Primer sequences**
GRMZM2G111611	Forward- ggtgttcgagggctacgag
	Reverse- ttggatctcggacagcttct
GRMZM2G003124	Forward- ggcactagggatgagagttca
	Reverse- cgctcactcaaaattagaacca
GRMZM2G085295	Forward- tatgcgcaactgcttcctc
	Reverse- catacccattccacccattg
GRMZM2G075496	Forward- aaaaagcaaagcgaaggaaa
	Reverse- gacgacgctaccaatcatcc
GRMZM2G010054	Forward- gggcagatgcttcaggtct
	Reverse- ggctctaactaccatggcttatct
GRMZM2G149406	Forward- ccatatgcgagcttgctg
	Reverse- ccatcgcttatctcgactgtg
GRMZM2G064993	Forward- catgccatggcaacgctcaaggtc
	Reverse- ctggttacctcactcctgccccaggag
GRMZM2G071292	Forward- ggtatgttgctgcattctgg
	Reverse- gccattttcatcctttgcag
Actin	Forward- gtgatggttggtatggggca
	Reverse- ctcagtcagcaacacagggt
